# Interleucina 35 (IL35): Um Novo Biomarcador para Doença Arterial Coronariana?

**DOI:** 10.36660/abc.20210991

**Published:** 2022-02-14

**Authors:** Gilson Soares Feitosa

**Affiliations:** 1 Escola Bahiana de Medicina e Saúde Pública Salvador BA Brasil Escola Bahiana de Medicina e Saúde Pública,Salvador, BA – Brasil; 2 Hospital Santa Izabel da Santa Casa da Bahia Salvador BA Brasil Hospital Santa Izabel da Santa Casa da Bahia,Salvador, BA – Brasil

**Keywords:** Doença da Arterial Coronariana, Interleucina 35, Biomarcadores, Diagnóstico por Imagem, Citocinas, Troponina

Em termos gerais, os biomarcadores são parâmetros biológicos associados à presença e gravidade de uma doença. Os biomarcadores podem ser detectados por diversos meios, incluindo o exame físico e a avaliação por exames laboratoriais ou por imagem.^1^

Uma definição mais recente e objetivamente direcionada restringe seus domínios a uma substância proteica *–* enzima, hormônio ou sem função conhecida *–* que se relaciona ao estado de saúde, indicando um bom estado de saúde ou doença, permitindo assim o diagnóstico de uma determinada doença, ou sua gravidade, ou então sendo um alvo terapêutico, ou servindo a finalidade de orientação terapêutica, que pode ser obtida a partir do sangue, urina, saliva ou outros fluidos ou tecidos corporais.^2^

No estudo publicado neste número do Arq Bras Cardiol. 2021, Oflar et al.,^3^ apresentam os resultados de uma avaliação caso-controle transversal dos achados da interleucina 35 (IL35) entre um grupo de 60 pacientes com doença arterial coronariana estável comprovada angiograficamente e um grupo de 46 indivíduos, também comprovada angiograficamente, sem doença arterial coronariana.

Eles afirmam ter demonstrado uma relação inversa entre o nível sérico de IL35 e a presença e gravidade da doença aterosclerótica coronariana. O Gensini e o Syntax Score medem a extensão.

Assim, eles sugerem que essa citocina anti-inflamatória parece estar reduzida em pacientes com doença arterial coronariana, principalmente na doença mais extensa.

O desenvolvimento de um biomarcador deve necessariamente seguir várias etapas, que começam pela identificação de um potencial candidato em uma fase pré-clínica, seguida de sua caracterização clínica *–* geralmente testando em um pequeno número de indivíduos, onde há o estabelecimento de valores de referência. Uma terceira etapa, geralmente com um grupo de controle, retrospectivo, um número modesto de participantes, serve para visualizar sua capacidade preditiva. Em seguida, vem a quarta fase, prospectiva, com um número consideravelmente maior de pacientes, onde se avalia a eficácia do teste e se constroem as curvas ROC. E, por fim, vem a fase cinco, longitudinal, com um número ainda maior de participantes, quando se estabelece a eficácia e aplicabilidade do teste.^4^

Este biomarcador ainda deve apresentar outras características exigidas: permitir medições repetidas e precisas, de preferência obtidas com rapidez e a um custo razoável.

Deve também fornecer informações e o que é possível obter com o exame clínico, sendo considerados para o estabelecimento das decisões clínicas.

Muitos biomarcadores potenciais estão sendo avaliados no momento atual, incluindo a perspectiva de sua aceleração de desenvolvimento devido à utilização progressiva da inteligência artificial.^5^

Na doença arterial coronariana, biomarcadores tradicionais têm sido intensamente utilizados em grupos distintos^6 , 7^ como a troponina e hs-CRP, e aqueles com insuficiência cardíaca, o BNP.^2^

O presente estudo é relevante porque reforça a ideia do papel potencial da IL35 neste contexto, como já foi considerado por outros,^8^ embora em um pequeno número de publicações.

Esperamos ver estudos subsequentes que irão testar este potencial biomarcador de forma mais definitiva em um estudo longitudinal adequado, prospectivo, com uma população maior e, esperançosamente, em grupos geográficos e étnicos distintos.
