# Supplementary material for: Interleucina 35 (IL35): Um Novo Biomarcador para Doença Arterial Coronariana?
Source: Arq Bras Cardiol. 2022 Feb 14;118(2):409–10. [Article in Portuguese] doi: 10.36660/abc.20210991 (PMC8856691; doi:10.36660/abc.20210991)
Supplement: Translation

# Translation: Interleukin 35 (IL35): A New Biomarker for Coronary Artery Disease?

**Authors:** Gilson Soares Feitosa

**Affiliations:** 1 Brazil Escola Bahiana de Medicina e Saúde Pública, Salvador, BA – Brazil; 2 Brazil Hospital Santa Izabel da Santa Casa da Bahia, Salvador, BA – Brazil

**Keywords:** Coronary Artery Disease, Interleukin 35, Biomarkers, Diagnostic Imaging, Cytokines, Troponin

In general terms, biomarkers are biologic parameters associated with an illness’s presence and severity. Biomarkers can be detected by several means, including the physical exam and evaluation by laboratory tests or by image.^1^

A more recent and objectively directed definition restricts its realms to a proteic substance – enzyme, hormone or without known function- that relates to health status, indicating a good status of health or disease, thus allowing for diagnosing a given disease, or its severity, or else being a therapeutic target, or serving the purpose of therapeutic guidance, that can be obtained from the blood, urine, saliva or other body fluids or tissues.^2^

In Study published in this issue of the Arq Bras Cardiol. 2021, Oflar et al.^3^ present the results of a transversal, case-control evaluation of the findings of interleukin 35 (IL35) between a group of 60 angiographically proven stable coronary artery disease patients and a group of 46 individuals, also angiographically proven, devoid of coronary artery disease.

They claim to have demonstrated an inverse relationship between IL35 serum level and the presence and severity of coronary atherosclerotic disease. The Gensini and the Syntax Score measure the extension.

Thus, they suggest that this antiinflammatory cytokine seems to be reduced in patients with coronary artery disease, mainly with the most extensive disease.

The development of a biomarker must necessarily follow several steps, which start by identifying a potential candidate in a pre-clinical phase, followed by its clinical characterization – usually testing in a small number of individuals, where there is the establishment of reference values. A third stage, usually with a control-group, retrospective, modest number of participants, serves to visualize its predictive capability. Then comes the fourth phase, prospective, with a considerably larger number of patients, where the efficacy of the test is evaluated, and ROC curves are built. And finally, comes phase five, longitudinal, with an even larger number of participants, when the effectiveness and applicability of the test are established.^4^

This biomarker still has to display other required characteristics: allow for repeated and accurate measurements, preferably obtained quickly and at a reasonable cost.

They must also provide information and what is possible to obtain by the clinical exam, being considered for establishment of clinical decisions.

Many potential biomarkers are being evaluated at the present moment, including the perspective of its acceleration of development because of the progressive utilization of artificial intelligence.^5^

In coronary artery disease, traditional biomarkers have been intensively utilized in distinct groups^6 , 7^ such as troponin and hs-CRP, and those with heart failure, the BNP.^2^

The present study is relevant because it adds to the idea of the potential role of IL35 in this context, as it has been considered by others,^8^ albeit in a small number of publications.

We look forward to seeing subsequent studies that will test this potential biomarker more definitively in a proper, prospective, longitudinal study, with a larger population, and hopefully in distinct geographical and ethnic groups.
